# Wherewithal Shall We Be Clothed?

**Published:** 1882-04

**Authors:** Ben. H. Pratt


### THE BISTOURY.

Vol. XIX. 18 ELMIRA, N. Y., APRIL, 1882. No. 1.

### Contributions.

### WHEREWITHAL SHALL WE BE CLOTHED?

BEN H. PRATT.

It has become the proper thing to prate upon our advanced civilization, and to boast thereof mightily. This would not seem so outrageous were the prater and boaster to hurry us away from the thought, and direct our minds elsewhere, without giving us time to think upon what our boasted civilization is founded. But when he who would impress upon our minds the truthfulness of his assertion, does so by pointing out, as a much vaunted effect of our lofty attainments in civilization, the fact that woman is revered in this age as she never has been in any other, and measures the height of our culture by this standard, then we begin to question whether the adoration paid to woman—amounting almost to deification—be not an index of our weakness rather then of our strength; of our folly rather then of our culture.

While it is repugnant to the instinct of the philanthropist of this age to deny that a certain degree of reverence for womankind is an index of a higher type of civilization, he must admit, if he think upon the question with any care, and without prejudice, that this boasted reverence is being carried too far, and that which we are pleased to term gallantry toward the female sex has been carried beyond the common sense point, and has lapsed into abject humiliation if not into absolute idolatry. It is one thing to assume a protective position as regards woman; to recognize her worth and weakness; to confess her our social equal and court her presence; to admit her ability to afford us enjoyment, not only in the higher vein of intellectual pastime, but also in the weightier; to carefully guard, honor and nurture her as the noblest being a wise Creator has provided for man's companionship; to provide for her needs as fully as we would provide for our own; to give her all the advantages we can bestow in the way of keeping her our equal in every social, moral and intellectual achievement, and to carefully avoid all those tendencies which naturally would result in creating and widening any breach between the sexes. It is another thing when our protection assumes the character of serfdom; when our recognition of her worth and weakness becomes worship; when we elevate her upon a higher and more ethereal plane than that upon which we stand; when we fawn in, rather than court her presence; when we metaphorically, if not actually, prostrate ourselves beher in tacit submission to her especial superiority, and suffer ourselves to be compromised by reason of our assumed inferiority; when we dare not deny her the possession of any object which her whim may demand and our effort may secure for her; when we nurture her as a divinity rather than as a nature like to our own; when we clothe her in a resplendence that unfits her for the position she was destined to fill, and makes her an object of curiosity rather than of respect and admiration; when we deck her with a jeweled fortune to arouse only the envy of her sex and the pity of our own; when we so carefully shield her from the cares and burdens of life as to make her a mere thing of ornament, of no use to any living being and the source of her own and our misery; and when in a word, we create between her and ourselves so great a gulf that companionship and mutual helpfulness is impossible.

That which I have indicated in the former clause *is* an index of rational civilization; that which I have only feebly portrayed in the latter, is not only the baldest folly, but it is one of the extremes of our modern school of culture, which is tending to destroy all the vaunted civilization of the age by making us sick of it, and arousing suggestions as to whether life would not be better with a return to semi-barbarism and some real social enjoyment, rather than to live along in this magnificent misery and soul solitude.

There is not room, in an article like this, to enter upon the many follies which this generation is becoming day by day more and more guilty of in the single direction of the treatment of women by men in what we are pleased to call good society. In the matter of dress alone, a paper of this sort is all too brief to more than suggest some of the absurdities we are encouraging, the follies we are nurturing, and the more potent results of these.

It is not possible that there is a man among my readers that has not more than once been compelled to note the vast difference between the sexes in the matter of attire alone. It has come to be acknowledged—more's the shame and the pity—that the wives and daughters of those who can afford to hire all the help they want in the domestic routine, must have nothing to do but study upon the designs of what they shall be attired in, and give themselves wholly to the task of making themselves beautiful in the eyes of those with whom they associate. All considerations are made subservient to this one end. We see, every day, the result. Men go to their business in the same suit of clothing from November to May, and in one other suit from May to November. It is of plain material, plainly cut, inexpressive, and worn without a thought as to what the impression it is making upon others may be. For occasions he may possibly have another suit, and in rare instances he may have still another, worn, perchance once or twice a season, under protest. Women appear in scores of different apparelment during the same season. This is the average, some in more, some in less, limited and gauged only by the monied ability of the providing power. Society, which is a mere aggregation of individuals in the same social standing, demands that the changes of feminine attire must not only be frequent, but each change must assure an expenditure of so much taste, so much thought, so much ingenuity and so much cash. There must be appurtenances thereto in the way of jewels, which may be worn oftener than once, but not too often. The ladies who thus appear in society are there for the purpose of displaying their wardrobes and the contents of their bijou drawer and jewel casket. They advertise these facts in their manner as well as in their dress. They seldom appear at ease, and are always exhibiting their self consciousness. They may not be continually looking at themselves directly, but they are constantly mirroring themselves by studying the impression they are making upon others. They learn to read the effect of their clothes upon the faces of those whom they meet, as well and as accurately as they have done the same before the two full length mirrors they posed between prior to their last adjustment of a skirt-fold and the final titivation of the rouge and powder appliances. They are occupied so exclusively with what they have on, and with the effect it is having upon others, that there is little if any room for anything in their minds except the receipt of homage. In the street, in the carriage, during the hasty call, in the boudoir, in the drawing-room, everywhere except at home during hours which society has not yet monopolized wholly, it is dress, dress, dress, and all the discomforts, the annoyances, the anxieties, the fears and the miseries thereto appertaining. Go she where she may, called forth, lured forth or driven forth by inclination, temptation, invitation or compulsion, the first and only question which at once forces itself upon her mind is, "Oh, dear! what shall I wear? How shall I wear it? What will be the effect of it?" There is worriment in the answer to the first, perplexity in that to the second, fear in that to the third, and misery in the combined result. Life, outside of a fashionable woman's duties to society, consists in studying the latest works on modes, and interviews with the most popular modistes. Home, husband, father, children, brothers, domestic duties, are all lost sight of in the one great and absorbing thought of what she will look best in, and what will most effectually sensationalize the society she moves in. This is her life, and this life she is tolerated in, if not encouraged to continue, by husband, father, brother, son and male associate. And what a life! To contemplate as present, to look forward to or back upon, O, shame upon us all, what a life!

And who is to be blamed for this? The woman? Yes, but not wholly. Society? Yes, but not alone. Men? Yes, but not entirely. None of these is conspicuously blameworthy in this bad business, this dreadfully mind-dwarfing characteristic of the age we live in, but either of these—woman, society, man—could, if it would, bring about a very different state of things.

Woman could effect a change by declaring herself disenthralled from the slavery she endures; by ignoring the dicta of society; by asserting her independence of Mrs. Grundy, and resolving that she will not longer be dependent upon polite opinion and the size of her provider's pocket-book. But this there are few women who are willing to do, and fewer still of those who are willing, are brave enough to attempt it. We certainly cannot expect any save the very strongest of them ever to do this thing. Their weaker heads are too full of vanity over the empty adulation they are constantly receiving from male admirers, to wish for a change. They have lived so long in the rarified air of compliment and flattery and tributes to their artificial charms, that the denser atmosphere of truth and outspoken good sense would shock them into a social death. The reform in dress, retrenchment from lavish expenditure of time and thought and money for appearances alone, must come from the bravest women of the land. We do not mean those of which Mary Walker is the type—bold women, who unsex themselves in their mad effort to revolutionize the relations between man and woman; but those who shall retain all their feminine graces, and yet conclude that they will not be outdone in the exhibition of common sense by men. What is needed is a combination of representative women who shall resolve that life has something higher in its aim than the effort to stun those with whom they associate, by the splendor of what they attire in; that to have a new robe for every party, a new hat for each month, a new set of jewelry for each quarter, and a small fortune per annum to expend upon dress alone, are extravagances which no wealth may warrant; that though it must needs be that there be butterflies, there need not be more of these among women than there are popinjays among men; that to be well and becomingly dressed is all that good taste and sound judgment demand; that if men can be—as they are—respected and honored for what they are rather than for what they wear, there is no reason why women should need any factitious charms to accomplish the same results; and that comfort, ease, health and happiness may all be secured and enjoyed by the sacrifice of a silly adoration of dress, for which women despise each other and are laughed at in secret by men who openly profess admiration therefor.

Society could materially assist women in their disenthralment from these worse than iron fetters, if it would. If any respectable number of those who move in so-called good society were to attach less importance than they do to what ladies appear in; if they ceased to compliment them upon their gorgeous appareling, and made it a point to commend for neat and substantial dressing rather than for ingenuity of mode and extravagance in expenditure; if it should chance to become the custom to think that it requires something else than dress to reach the popular ideal of ladyhood; if a realizing sense of the folly of burdening the feminine creation with the discomforts of over dressing, and of taxing their entire time and mental resources with all the anxiety necessary to accomplish such miraculous toilets; there would soon be a modification of the evil, if nothing more.

But if, in addition to these, men who really love their wives and their daughters and their sisters were to kindly say to them that they are not advancing their charms in the eyes of sensible men by their efforts to outdo each other in the marvel of toilet-making, and in addition thereto would firmly insist upon it that there are nobler uses to which thought and money could be applied than by selfishly heaping the body with costly raiment, it seems to me that the innate good sense of woman could be led to assert itself in the direction of such a change, such a modification in the degree of dress, as to restore woman to something more than a lay figure to display costly apparel upon.

There is something noble in a simply dressed woman, one who is attired in a manner befitting the occasion. It will not be demanded that she discard all species of adornment and return to the plain garb we are accustomed to greet our Quaker friends in, but that she consult the proprieties rather than the sensational, and do not consider herself tabooed if she appear thrice or oftener in the same toilet upon even the most brilliant social occasions; that it will be to her credit rather than otherwise, to so dress that she may not be conspicuous either by reason of her fine or plain dressing; that they who meet her may not be able to say precisely what she had on because of its unpronounced character; in a word, to make dress not even a secondary consideration in life, but subordinate to many another. I hope to see the time when I may count among my best friends a coterie of women who will not worry inordinately over the matter of dress; who will purchase their cold weather suits and their warm weather suits of clothing just as men do, and wear them just as men do—

put them on when they rise in the morning, wear them all day and every day until they are no longer presentable, and stop all this senseless, time-stealing, happiness-destroying custom of changing the dress twice, thrice or oftener between the morning toilet and the retiring hour. Only for those who do manual or menial labor is there any excuse for a change of garments other than as I have noted. There are no more grades of occupation among women than among men, and as it would be manifestly improper for a laboring man to spend the evening with his family or among his associates with the soiled clothes he brought from his day's toil, so it would be improper for the ladies of the household to appear in the same toilets they busied themselves about the kitchen in, or wore upon sweeping days. Let the toiler, man or woman, consult decency, and change the attire for the evening's better enjoyment, but those ladies who seldom, if ever, do aught to soil a sensibly chosen dress, may appear, and should be encouraged to appear, in society in a day dress, and might do so as properly as men spend their evenings at informal social gatherings in business suits. If one elicits no remark of surprise, neither should the other; and as, upon more formal occasions, men are sometimes tempted into a semi, and again into a full dress suit, the same privilege might reasonably be accorded to women. But as there is no occasion upon which more men wear their very oldest suits than they do at what is termed "a full dress" affair—for a man's dress suit, doeskins, claw hammer, light kids and all, are fairly musty with their long coffinage in his bureau drawer or clothes press—and yet he is none the worse in reputation for having worn it, though he may despise himself for his temporary weakness in making a monkey of himself in his loyalty to a social custom as discomforting as it is ridiculous. As he is as good as ever, when he gets around into his accustomed habiliments, so why may not a woman fish out from among her wardrobe her one handsome dress and wear it without a grimace for the fifty-ninth time. For the life of me I cannot see any reason for the grand distinctions they draw between men and women in this matter of dress.

As I before said, there will of necessity be butterflies and popinjays, but let not the sensible folk in society be among them. To be well dressed and not overdressed should be one of those matters in life worth some attention; but there are scores of considerations to which it should be subordinate, both in men and women. The subject of dress is getting to be so formidable an one that it usurps everything else, and from the rich deacon's wife in the best pew to the poor domestic away back under the organ loft, it counts more than the plan of salvation, and it's time something were done about it. We men don't pretend to be models in much, but in sensible dressing we rather plume ourselves.

